# Sign of Leser-Trélat with an adenocarcinoma of the prostate: a case report

**DOI:** 10.4076/1757-1626-2-8868

**Published:** 2009-08-26

**Authors:** Nicolas Kluger, Bernard Guillot

**Affiliations:** Service de Dermatologie, Universite Montpellier I, Hopital Saint-EloiCHU de Montpellier, 80, Avenue Augustin Fliche, FR-34295 Montpellier Cedex 5, France

## Abstract

**Introduction:**

The sign of Leser-Trélat is defined by the sudden appearance and rapid increase in number and size of seborrheic keratoses, preceding or revealing a malignancy. Even though this sign remains controversial, it has been described during a wide range of malignancies, including mainly adenocarcinoma of the gastro-intestinal tract or the breast.

**Case presentation:**

We report the case of a 68-year-old man who experienced sudden increased in number of seborrheic keratoses within two years prior to a diagnosis of adenocarcinoma of the prostate. After prostatectomy, pigmented lesions stopped their brutal increase but did not regress.

**Conclusion:**

This is the second case of adenocarcinoma of the prostate associated with the sign of Leser-Trélat. This report acts as a reminder that in case of Leser-Trélat sign, a complete physical examination is mandatory followed by specific complementary exams.

## Introduction

The sign of Leser-Trélat is defined by the sudden appearance and rapid increase in number and size of seborrheic keratoses, preceding or revealing a malignancy [1]. Even though this sign remains controversial, it has been described during a wide range of malignancies, including mainly adenocarcinoma of the gastro-intestinal tract or the breast [1]. We report here the second case of adenocarcinoma of the prostate associated with the sign of Leser-Trélat [2].

## Case presentation

**Figure 1.**
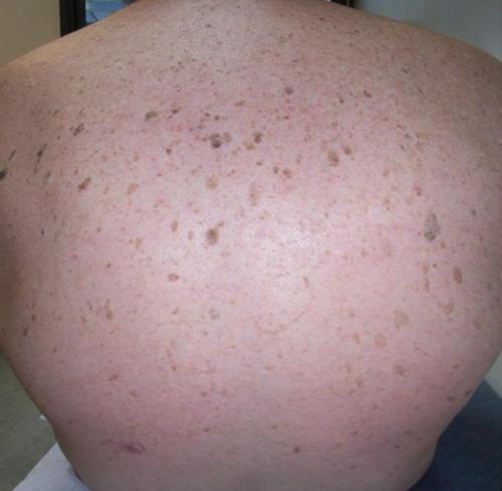
Seborrheic keratoses of the back displaying a rain drops pattern.

A 68-year-old French man of Caucasian origin presented for the treatment of pigmented lesions he had for several years. Physical examination disclosed numerous sharply demarcated, verruca-like, hyperpigmented lesions of the trunk and the back, with a typical “rain drops” or “splash” pattern (Figure 1). Other lesions were noted on the upper and lower limbs as well as a few ones on the face. Lesions were all clinically consistent with seborrheic keratoses. His medical history was notable for an adenocarcinoma of the prostate treated by prostatectomy 5 years ago. When interviewed, the patient spontaneously mentioned that he had noticed a link between prostate cancer and the skin lesions with a sudden increase in number during the two years prior to diagnosis. Indeed, the patient did acknowledge the presence of this type of lesions for years before cancer diagnosis. Nevertheless, during the two preceding years, he presented clinical dysuria associated with benign prostatic hypertrophy on ultrasonography and normal seric prostate specific antigen (PSA) and, meanwhile, he experienced explosive increase of seborrheic keratoses. After two years, prostate biopsies, performed because of an elevation of PSA, disclosed an adenocarcinoma. After prostatectomy, seborrheic keratoses stopped their brutal increase but did not regress. The patient still presents generalized seborrheic keratoses with gradual onset but lesions tend to occur with a lower rate.

## Discussion

The sign of Leser-Trélat has been described with a wide range type of cancer including mostly adenocarcinoma and may be related to a tumor-secreted growth factor [1]. There is no precise definition of the length of the eruptive period and the number of keratoses that are needed for the diagnosis [1]. This sign remains however controversial as both cancer and seborrheic keratosis are common in elderly patients. Moreover, distinguishing eruptive seborrheic keratoses as defined by the sign of Leser-Trélat and “benign” seborrheic keratoses of gradual onset is often challenging, especially on the basis of the patient interview [1]. In our case, the patient mentioned spontaneously that the lesions occurred suddenly before the diagnosis of prostate cancer during the consultation before he was asked any question. He did not deny he had seborrheic keratoses before the urinary symptoms occurred or new lesions after surgery, but he noticed that the onset of the lesions was then way slower.

This is to date the second case of prostate cancer associated with the sign of Leser-Trélat [2]. As suggested by Schwartz, complementary exams are subjected to clinical findings [1]. Our case acts as a reminder that in case of Leser-Trélat sign a complete physical examination is mandatory and any clinical anomaly should prompt targeted complementary exams.

## Abbreviation

PSA: prostate specific antigen
